# How to Choose a Doctor

**Published:** 1870-10

**Authors:** Thos. K. Beecher


### HOW TO CHOOSE A DOCTOR.

THOS. K. BEECHER.

We have read somewhere about "how to choose a wife," somewhere else about how to buy a horse," and somewhere, too, "how to write a letter," or "make a garden," or "cure the rot in sheep,"—yes, we have read "how to get rich," and how to do every thing under the sun, except HOW TO CHOOSE A DOCTOR. Since we cannot find an article about how to choose a doctor, we will e'en write one.

To be a doctor, one must first be a man, and a mean man cannot be a good doctor anymore than he can be a good minister or a good husband. And a really honest, large and loving man cannot make a poor doctor, no matter what his pet *pathy* may be. To have good sense as a doctor one must have good sense as a man. If your doctor is a nincompoop about other things, you may be sure that he is a ninny as to medicine and surgery.

If the doctor's office be untidy and vile to smell of, you may be quite certain that he will come short of giving good council as to health and tidiness of body. If he be clumsy in hitching his horse, you may be sure that he is not handy at surgery or midwifery. If he be a great, coarse, blundering fellow—careless of dress, a two fisted, farmer looking man, you may be sure that he will lack perception of those finer symtoms by which a good doctor is guided.

If he slanders brother physicians, who profess a different pathy, you may be sure that he is himself a quack. Good, earnest doctors are too busy to find time to slander their brethren or their rivals. It is all the same with lawyers, ministers and teachers. The truly good and truly great do not detract from the reputation of others; they are generous and magnanimous even to rivals.

If your doctor flatters you and humors your lusts and appetites, and helps you out of a bad scrape secretly, without reproof as if you had done no wrong, distrust him. If you can hire him to do or say what he would not do without the hire, beware of him. Good doctors cannot be bought. Your doctor ought not to be a single man. He ought to have a wife and children, and if you see that his wife respects him and his children obey him, that is a very good sign that he may be trusted.

If your doctor tells you how to *keep* well, that is a good sign. You come to him with toothache, and he gives you kreosote and clove oil for the tooth, and at the same time suggests that you do not wash enough to keep well—that is a good sign.

If the children like him, that is a sign. If you find him reading in his office, that is a good sign, and specially, if he be a settled, middle aged man. If you hear him say, "I once thought" so and so, but "I was wrong," that is a good sign.

If the doctor is neat and handy in rolling pills and folding powders, that is to his credit as a surgeon.

If he understands how to bud roses, graft fruit trees, mix strawberry pollen for improved berries, cure chicken pip, and tinker a trunk-lock, or put a clock in order, all these are so much to his credit.

If further, you love to meet him, the sight of him quickens you, and you are glad to hear him chat; and you know him thus to be a lovable, sympathetic man—he's the man for your doctor, your confidential friend,—find him—trust him.

FOWLS VERSUS WORMS.—M. Giot, a French entomologist, has lately found new employment for fowls. He says that French farmers have, during the past year, complained bitterly of the prevalence of worms, which infest corn and other crops, the highest cultivated fields being the most infested. Fowls are known to be the most indefatigable worm destroyers, pursuing their prey with extraordinary instinct and tenacity. But fowls cannot conveniently be kept upon every field, nor are they wanted there at all seasons. Therefore M. Giot has invented a perambulating fowl-house, which is described as follows; "He has large omnibuses, fitted up with perches above, the nest beneath. The fowls are shut in at night, and the vehicle is drawn to the required spot, and the doors being opened every morning, the fowls are let out to feed during the day in the fields. Knowing their habitation, they enter it at nightfall without hesitation, roost, and lay their eggs there."

